# Editorial: Spatiotemporal heterogeneity in CNS tumors

**DOI:** 10.3389/fimmu.2024.1430227

**Published:** 2024-05-29

**Authors:** Syed M. Faisal, Vidhya M. Ravi, Jason M. Miska

**Affiliations:** ^1^ Department of Pediatrics, Division of Hematology/Oncology, Children’s Mercy Research Institute, Kansas City, MO, United States; ^2^ Department of Neurosurgery, Medical Center – University of Freiburg, Freiburg, Germany; ^3^ Lou and Jean Malnati Brain Tumor Institute, Robert H. Lurie Comprehensive Cancer Center of Northwestern University, Chicago, IL, United States

**Keywords:** glioblastoma, spatiotemporal heterogeneity, spatial transcriptomics, multiplex spatial 3D mapping, single cell spatial analysis

Central nervous system (CNS) tumors, particularly gliomas such as glioblastoma (GBM), have the worst prognosis. The median survival time was only 14–16 months. This is largely due to their immunosuppressive tumor microenvironment (TME) and pronounced heterogeneity at the spatial, temporal, cellular and molecular levels (1–3). The intricate crosstalk of cellular and molecular spatiotemporal heterogeneity within these tumors presents significant challenges for developing effective treatment strategies (2–4).

Recent advancements, such as the introduction of spatially resolved T cell receptor sequencing (SPTCR-seq) by Benotmyane et al., highlight the rapid evolution of our analytical methods. This technique utilizes optimized target enrichment and long-read sequencing to surpass existing technologies in reconstructing complete TCR architectures and evaluating T cell diversity in cancer. This progress advances our understanding of cancer immunosuppression and signals a broader trend in the field. In addition to spatial analysis, new methodologies are emerging, driving us toward significant clinical benefits. As these technologies continue to evolve, we are optimistic that the coming years will see these innovations translate into noticeable improvements in patient outcomes, indicating that we are moving in the right direction (5).

Gene therapy is experiencing a resurgence, highlighted by the recent approval of Todo’s oncolytic HSV-1 G47Δ gene therapy in Japan. This marked a significant milestone in the treatment of residual or recurrent GBM, where Todo et al.’s phase I and II clinical trials have demonstrated promising results, including encouraging one-year survival rates. These trials incorporated histopathological analysis of matched samples taken from primary surgery before treatment and from secondary surgery posttreatment, revealing enhanced recruitment of immune cells in the secondary tissue following gene therapy (6, 7). Furthering this line, Umemura and coauthors employed multiplex spatial analysis in their phase 1 clinical trial to map the distribution of tumor cells, immune cells, and other cellular constituents within the glioma microenvironment. Their findings suggested a significant increase in the immune region characterized by a high density of CD45^+^ lymphocytes in six out of eight patients following dual viral vector Ad-TK and Ad-Flt3L-based gene therapy (8). Conversely, the tumor region, identified by a high density of SOX2^+^ tumor cells, showed a decrease in all patients. Interestingly, the analysis revealed that immune cells are often entrapped by myeloid cells in the cellular neighborhood, rendering them ineffective. The study also highlighted a lack of investigations into the ‘other cell’ region, which is marked by a high density of CD45^-^ and SOX2^-^ cells. This area, potentially comprising neurons, reactive glia, and myeloid-derived suppressor cells, could be pivotal in tumor recurrence and resistance, emphasizing the need for further spatiotemporal research in this crucial area (8).

The emphasis on spatiotemporal heterogeneity in this editorial is particularly relevant and timely. As brain tumors evolve, they exhibit distinct patterns of gene expression and cellular behavior, influenced by their spatial localization within the tumor mass. This spatiotemporal heterogeneity complicates treatment strategies and often leads to therapeutic resistance and recurrence (**Figure 1**). The Research Topic titled “*Spatiotemporal heterogeneity in CNS tumors*” includes five pioneering research and review articles that utilize advanced spatial biology techniques to decode the complex landscape of brain tumors. Each article significantly advances our understanding of spatiotemporal tumor dynamics, offering insights that could lead to guide the development of more targeted and effective therapeutic interventions.

**Figure 1:**
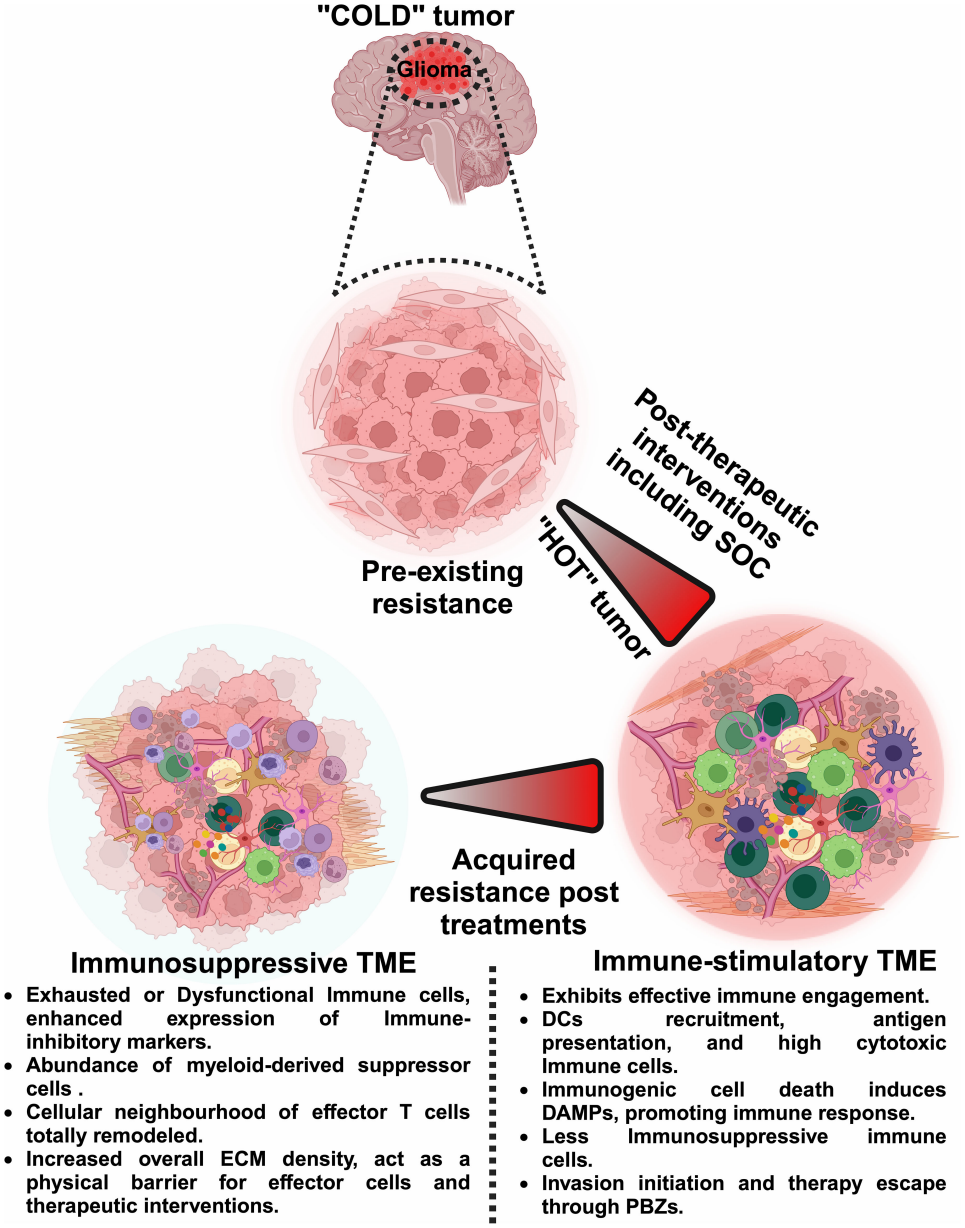
Dynamic shifts in the glioma microenvironment: from ‘Cold’ to ‘Hot’ tumors. This figure illustrates the dynamic shifts within the glioma TME, comparing the immunologically ‘cold’ tumor states characterized by pre-existing resistance with the ‘hot’ tumor states that arise post-therapeutic interventions. It delineates how ‘cold’ tumors, which exhibit minimal immune activity and inherent resistance, can transition to ‘hot’ tumors, which are more responsive to immune interventions. However, these ‘hot’ tumors can revert to ‘cold’ states due to acquired resistance, emphasizing the need for spatiotemporal analysis. Such analyses, including single-cell transcriptomics, multiplex immunohistochemistry, imaging mass cytometry, *in vivo* time-lapse microscopy, and 3D mapping, are essential to continuously adapt and optimize therapeutic strategies. By precisely mapping the temporal and spatial immune landscape, neurooncologists can better manage treatment protocols and enhance the efficacy of interventions against glioma. Image was created with BioRender.com.

The articles in this Research Topic address the complexities of CNS tumors by offering novel insights into their spatial and temporal dynamics, thereby suggesting strategies to overcome the challenges posed by the immunosuppressive glioma TME. For instance, a detailed review article by Shireman et al. highlighted advanced imaging and spatial transcriptomics to map gene expression variations across different tumor regions. Spatiotemporal analysis implementation elucidates molecular signatures associated with tumor resistance and aggressiveness and underscores the potential of spatial technologies to identify new therapeutic targets by capturing the heterogeneity within tumors.

Another pivotal theme that emerges from the Research Topic is the interaction between tumor cells and the immune system, particularly through the CXCR6/CXCL16 axis in T-cell and myeloid cell interactions within the glioma TME. In a seminal contribution, Chia et al. explored how CNS tumors manipulate their microenvironment to evade immune surveillance by employing spatial analysis to pinpoint regions of immune cell exclusion and immunosuppression​​. This article paves the way for developing immunotherapeutic strategies tailored to circumvent the immunosuppressive nature of GBM. This study further demonstrated that CXCR6 expression is essential for T-cell activation and migration to the brain, yet it may also contribute to T-cell dysfunction within the glioma TME, illustrating the dual role of this pathway in regulating antiglioma immunity.

Ballestin et al. in their article, elaborate on the unique biological and clinical characteristics of the peritumoral brain zone (PBZ). These findings highlight the potential for localized therapeutic strategies and the need for a deeper understanding of the PBZ zone, an area extending several centimeters around the tumor core. This zone harbors specific molecular, radiological, and cellular interactions that influence glioma cell proliferation and invasion and is crucial for effectively combating tumor recurrence.

Hou et al. also explored the spatiotemporal heterogeneity of B, T, and myeloid cells within the glioma TME. They investigated the potential of B cells in enhancing the efficacy of immunotherapies against GBM. They developed a novel vaccine formulation, BVax, which leverages B cell functions activated by the CD40 agonist, IFNγ, and BAFF to generate a potent anti-GBM B-cell vaccine. This vaccine demonstrates enhanced anti-presenting potential compared to that of naive B cells and even dendritic cells. These findings suggest that BVax improves T cell activation and promotes the proliferation of stem-like memory T cells, which are crucial for sustained antiglioma immunity. This approach holds promise for developing more effective treatments for glioma and potentially other types of cancer, highlighting the dual functionality of BVax in driving robust immune responses while overcoming the immunosuppressive nature of the glioma TME.

Collado et al. discusses the role of the extracellular matrix (ECM) in modulating immune responses within the GBM TME, illustrating how ECM-associated proteins inhibit the efficacy of immunotherapies through complex interactions with tumor and immune cells. Understanding the impacts of these proteins, their perspective highlights their significant roles in tumor growth and immune cell infiltration. Previously, we demonstrated that COL1A1 serves as an actionable target to disrupt glioma progression through spatiotemporal analysis of glioma heterogeneity (9). We also developed an *in vitro* model to study glioma dynamics (10). The authors propose that manipulating the ECM could enhance immune cell infiltration and consequently, the efficacy of immunotherapy, opening new avenues for treatment strategies.

This Research Topic brings together cutting-edge studies that employ spatial biology to delve into the depth of GBM heterogeneity, offering a comprehensive view of the complexities of glioma. From detailing the peritumoral brain zones to exploring the implications of immune cell infiltration and cellular plasticity, the contributions within this Research Topic offer a comprehensive overview of the current landscape and suggest promising future directions in glioma research. Each article not only advances our understanding of spatiotemporal tumor dynamics but also emphasizes the potential of spatial transcriptomics as a beacon for the development of novel therapeutic strategies and combinational treatments aimed at overcoming therapeutic resistance in glioma. As we continue to explore these innovative approaches, we will move closer to a future where our enhanced understanding translates into significantly improved outcomes for patients.

## Author contributions

SF: Conceptualization, Data curation, Investigation, Writing – original draft, Writing – review & editing. VR: Conceptualization, Data curation, Investigation, Writing – review & editing. JM: Conceptualization, Data curation, Investigation, Writing – review & editing.
